# The effect of a pelvic floor training program on perineal trauma during birth: a patient-preference controlled clinical trial

**DOI:** 10.1038/s41598-026-47603-6

**Published:** 2026-04-06

**Authors:** Mehmet İncebıyık, İsmail Palalı, Yasemin Er, Ömer Tammo, Yusuf Ziya Kızıldemir, İbrahim Halil Adak, Rahime Kada Düken

**Affiliations:** 1 Department of Obstetrics and Gynecology, Harran University Faculty of Medicine, Şanlıurfa, Türkiye; 2 Department of Physiotherapy, Harran University Faculty of Health Sciences, Şanlıurfa, Türkiye; 3 Department of Physical Medicine and Rehabilitation, Mehmet Akif İnan Training and Research Hospital, Şanlıurfa, Türkiye; 4 Department of Obstetrics and Gynecology, Şanlıurfa Training and Research Hospital, Şanlıurfa, Türkiye

**Keywords:** Pelvic floor muscle training, Perineal trauma, Patient-preference controlled trial, Episiotomy, Maternal outcomes, Diseases, Health care, Medical research, Urology

## Abstract

Pelvic floor muscle training (PFMT) is widely recommended for the prevention of urinary incontinence, but its potential to prevent perineal trauma during childbirth remains uncertain. We investigated whether a structured antenatal PFMT program could reduce severe perineal trauma and positively influence selected obstetric and neonatal outcomes. This prospective, non-randomized, patient-preference controlled trial was conducted at two tertiary centers between December 2, 2024, and September 2, 2025. Low-risk nulliparous women (defined as singleton, cephalic pregnancy at term without medical complications) at ≥ 28 gestational weeks were invited to participate. Those who consented formed the PFMT group, and those who declined received standard antenatal care. The intervention consisted of twice-weekly supervised PFMT sessions plus daily home exercises (3 sets of 8–12 maximal contractions) until 34 weeks of gestation. To minimize detection bias, clinicians assessing perineal outcomes were blinded to group allocation. The primary outcome was severe perineal trauma, defined as third- or fourth-degree obstetric anal sphincter injury (OASI). Secondary outcomes included episiotomy rate, first- and second-degree perineal tears, duration of the second stage of labor, postpartum urinary incontinence, and 5-minute Apgar score < 7. Between-group comparisons were performed using t-tests or chi-square tests, with effect sizes and 95% confidence intervals reported. Of 358 women screened, 300 completed the study (PFMT *n* = 150, Control *n* = 150). Severe perineal trauma was significantly lower in the PFMT group (4.0% vs. 14.7%; OR 0.24, 95% CI 0.09–0.64, *p* = 0.009). The second stage of labor was significantly shorter in the PFMT group (49.7 ± 6.4 vs. 58.8 ± 20.4 min, *p* < 0.001). No significant between-group differences were observed regarding secondary outcomes, including episiotomy rates (*p* = 0.157), postpartum urinary incontinence (*p* = 0.645), or adverse neonatal outcomes. Structured antenatal PFMT may reduce severe perineal trauma and shorten the second stage of labor without compromising neonatal outcomes. These findings support the integration of PFMT into antenatal care, although larger randomized trials with long-term follow-up are warranted to confirm these results.

## Introduction

Perineal trauma is one of the most common complications of vaginal delivery, affecting up to 85% of primiparous women, and includes both spontaneous lacerations and episiotomies of varying severity^1,2^. Severe forms, such as third- and fourth-degree obstetric anal sphincter injuries (OASI), are associated with significant short- and long-term morbidities, including chronic pelvic pain, dyspareunia, urinary and fecal incontinence, and pelvic organ prolapse, all of which negatively affect postpartum quality of life^3,4^. Therefore, preventing perineal trauma is an important obstetric goal.

Multiple maternal and obstetric risk factors have been identified, including primiparity, prolonged second stage of labor, instrumental delivery, fetal macrosomia, and insufficient perineal support^5,6^. Non-invasive preventive strategies are increasingly emphasized, among which antenatal pelvic floor muscle training (PFMT) has gained attention. PFMT aims to improve pelvic floor strength, elasticity, and neuromuscular control, which is hypothesized to facilitate more controlled pushing and support fetal descent, potentially reducing the risk of perineal injury during birth^7^. Increased PFM strength and enhanced neuromuscular coordination are hypothesized to improve the structural integrity and functional adaptation of the perineal tissues. Specifically, better neuromuscular control may facilitate more effective relaxation and controlled stretching of the pelvic floor during the crowning of the fetal head. This allows the tissues to better accommodate the significant mechanical distension encountered during the second stage of labor, thereby potentially reducing the incidence of high-grade spontaneous lacerations^1,2,6^.

Several randomized controlled trials and systematic reviews have investigated the effectiveness of PFMT in pregnancy. A systematic review and meta-analysis by Du et al. (2015) reported that antenatal PFMT shortened the second stage of labor and reduced episiotomy rates, but evidence on perineal trauma reduction was inconsistent^8^. More recently, a 2020 systematic review and meta-analysis by Sobhgol et al. confirmed that PFMT can positively influence labor outcomes but highlighted heterogeneity across studies and called for further well-designed trials^9^. The 2020 Cochrane review on antenatal PFMT concluded that while PFMT is highly effective for preventing urinary incontinence, its role in preventing perineal trauma remains uncertain, with insufficient high-quality evidence from diverse populations^10^.

Importantly, most of the existing studies were conducted in Western countries, and their findings may not be directly generalizable to populations with different socio-cultural backgrounds, ethnic variations, and obstetric practices related to pregnancy and childbirth. Studies have suggested that anthropometric characteristics and tissue elasticity may vary across ethnic groups, potentially influencing the risk of laceration and the response to PFMT^11,12^. Additionally, cultural differences in daily physical activity levels and acceptance of exercise programs during pregnancy may affect adherence and outcomes^13^.

Episiotomy practice varies widely across countries and settings, and rates may be substantially higher in some regions and hospital types. In Türkiye, episiotomy has historically been used more frequently in routine practice, particularly in high-volume tertiary centers, which may influence the overall distribution of perineal outcomes in observational cohorts^14^. Importantly, the World Health Organization recommends restrictive rather than routine use of episiotomy and notes that population episiotomy rates should generally not exceed approximately 10%. Given this context, reporting episiotomy rates and interpreting perineal outcomes requires careful consideration of local obstetric practice patterns^15^.

### Study aim and hypothesis

This study aimed to evaluate the effectiveness of a structured, supervised Pelvic Floor Training Program (PFTP) in reducing perineal trauma and improving maternal and neonatal outcomes among low-risk nulliparous women. We hypothesized that participation in a structured PFTP would reduce the incidence of severe perineal trauma (third- or fourth-degree tears), shorten the second stage of labor, and improve overall maternal birth outcomes without compromising neonatal safety.

### Contribution to the literature

This study provides region-specific data from a Middle Eastern population and may help determine whether PFMT should be included in routine antenatal care protocols in similar settings. By using a patient-preference controlled design and a standardized, supervised intervention protocol, our study adds to the growing body of evidence on the role of PFMT in obstetric care.

## Materials and methods

### Study design and Setting

This was a prospective, patient-preference controlled clinical trial conducted between December 2, 2024, and September 2, 2025, at Harran University Research and Training Hospital and Şanlıurfa Training and Research Hospital, two tertiary referral centers in southeastern Türkiye. The study protocol was approved by the Harran University Clinical Research Ethics Committee (Approval No: HRÜ/24.19.31) and was conducted in accordance with the Declaration of Helsinki. Written informed consent was obtained from all participants.

This trial was registered on ClinicalTrials.gov (NCT07292948, registration date: 18/12/2025). Although registration was completed retrospectively after enrollment had started due to administrative delays, we clarify that the primary and secondary outcomes were strictly predefined before participant recruitment began. Furthermore, the study followed the initial research plan without any protocol deviations regarding intervention delivery or outcome assessment, thereby ensuring methodological transparency.

A patient-preference design was utilized to enhance participant adherence and reflect real-world clinical effectiveness, as mandatory randomization in exercise interventions can lead to high attrition or group contamination .

### Participants

Low-risk nulliparous women were screened and counseled beginning at 20 gestational weeks to allow scheduling. Formal study enrollment (group allocation) and the start of supervised PFMT occurred at ≥ 28 gestational weeks; only women enrolled at ≥ 28 weeks were included in the analysis. Gestational age at enrollment was summarized for each group and reported as mean ± SD.

To ensure a homogeneous study population, the following criteria were applied to define low-risk pregnancy:

Inclusion criteria consisted of: (1) nulliparous women; (2) singleton pregnancy; (3) cephalic presentation at term; and (4) age between 18 and 40 years.

Exclusion criteria were: (1) pre-existing or gestational medical complications (e.g., pre-eclampsia, gestational diabetes requiring insulin, or chronic hypertension); (2) suspected fetal macrosomia (estimated fetal weight > 4000 g); (3) prior pelvic, vaginal, or perineal surgery; (4) body mass index (BMI) at enrollment > 35 kg/m²; and (5) any physical or medical contraindications to exercise.

Participants in the control group provided informed consent for the collection and analysis of their clinical data but specifically chose not to participate in the structured exercise intervention (patient-preference).

### Intervention: pelvic floor training program (PFTP)

The PFTP was developed in accordance with the Consensus on Exercise Reporting Template for Pelvic Floor Muscle Training (CERT-PFMT) and the Template for Intervention Description and Replication (TIDieR) guidelines to ensure high reproducibility and methodological rigor^16^. During supervised sessions, correct pelvic floor muscle (PFM) activation was verified using standardized verbal instruction and palpation/observation to identify substitution patterns. If participants demonstrated Valsalva/bearing down, gluteal or abdominal co-contraction, or delayed/absent relaxation between contractions, the physiotherapist provided immediate cueing and modified the task (shorter hold duration, longer rest periods, emphasis on breathing and full relaxation) until an isolated PFM contraction–relaxation pattern was achieved. Participants who could not perform an isolated contraction received additional one-to-one coaching before progressing to the full protocol.

### Supervised sessions

The supervised sessions were led by a pelvic floor–certified physiotherapist (PhD, PT) with expertise in obstetric pelvic floor rehabilitation. Women identified during early antenatal screening were informed about the program from 20 gestational weeks; however, the supervised PFMT intervention formally commenced only after study enrollment at ≥ 28 gestational weeks and continued until 34 weeks, providing at least 6 weeks **of** supervised training. Sessions were conducted twice weekly in an outpatient clinical setting. Each session lasted approximately 20 min and consisted of three sets of 8–12 voluntary pelvic floor muscle contractions held for 6–8 s, with equal relaxation intervals between contractions to minimize fatigue. Participants received real-time verbal feedback to ensure correct technique and to avoid Valsalva/bearing down or accessory muscle co-contraction.

### Technique and progression

Correct technique was monitored through verbal cueing and digital vaginal palpation. Progression was achieved by increasing training load (longer holds, more repetitions/sets, shorter rest intervals, more challenging positions) and by improving contraction quality and endurance, rather than attempting to exceed a maximal voluntary contraction. If incorrect patterns were observed such as Valsalva maneuvers, breath holding, or co-contraction of gluteal or adductor muscles immediate corrective feedback was provided until proper “lift and squeeze” activation and full relaxation were achieved.

### Home exercise program

Participants were instructed to perform a daily home-based regimen of three sets of 8–12 contractions on non-supervised days to maintain training continuity.

### Adherence monitoring

Attendance at supervised sessions was documented, and adherence to home exercises was monitored through weekly follow-up phone calls and participant-completed exercise diaries submitted during clinic visits.

### Control group

The control group received standard antenatal care without structured PFMT sessions. Routine advice on general physical activity was permitted in accordance with standard clinical practice.

### Outcome measures

The **primary outcome** was the incidence of severe perineal trauma, defined as third- or fourth-degree obstetric anal sphincter injuries (OASI). Diagnosis was performed immediately after delivery by the attending obstetrician or midwife.

**Secondary outcomes** included episiotomy rate, first- and second-degree perineal tears, duration of the second stage of labor, postpartum urinary incontinence, and 5-minute Apgar score less than 7.

**Labor and delivery outcomes**:

- Episiotomy rate.
- Duration of the second stage of labor, measured in minutes from full cervical dilation to fetal expulsion.

### Postpartum urinary incontinence (UI)

Postpartum urinary incontinence (UI): UI was assessed at 6 weeks postpartum during a scheduled in-person clinical follow-up visit (± 7 days). UI was defined as any self-reported involuntary urine loss within the preceding week. For structured assessment, we used the validated Turkish version of the Pelvic Floor Distress Inventory-20 (PFDI-20). Presence of UI was recorded when participants reported any non-zero symptom on the Urogenital Distress Inventory (UDI-6) subscale.

**Neonatal outcomes**:

- Birth weight.
- 1- and 5-minute Apgar scores, with the 5-minute score categorized as < 7 or ≥ 7.

### Data collection and blinding

Baseline demographic and clinical variables (maternal age, weight, and educational level) were collected at enrollment. Labor and delivery variables were extracted from standardized institutional delivery records.

To ensure the integrity of blinding in routine clinical practice, group allocation was managed by the research physiotherapists and was not recorded in the patients’ primary obstetric medical charts used during labor and delivery. The clinicians and midwives who managed the deliveries and assessed the perineal outcomes were independent of the research team and were not informed of the participants’ group allocation. Communication between the physiotherapists delivering the intervention and the obstetric staff was strictly limited to ensure that the birth attendants remained unaware of whether a patient had undergone the structured PFMT program or was in the control group. This separation between the intervention delivery and the clinical assessment of outcomes was maintained throughout the study to minimize detection bias.

### Sample size calculation

The sample size was calculated to detect a 50% relative reduction in severe perineal trauma, assuming a baseline incidence of 15%, with α = 0.05 and 80% power. This yielded a required sample size of 270 participants. To account for potential attrition, we aimed to recruit 318 women (PFMT *n* = 161; control *n* = 157). After 18 losses to follow-up, 300 participants (150 per group) were included in the final analysis.

### Statistical analysis

Data were analyzed using SPSS version 22. Continuous variables were assessed for normality using the Shapiro–Wilk test. Given the relatively large sample size, the interpretation of normality testing was supplemented by visual inspection of histograms and Q-Q plots to ensure a robust assessment of data distribution. Continuous variables were compared with the independent-samples t-test or Mann–Whitney U test, as appropriate. Categorical variables were compared using the chi-square test or Fisher’s exact test.

To identify the independent effect of PFMT on the primary outcome (severe perineal trauma), a multivariable logistic regression model was performed. The covariates included in this model—maternal age, BMI, and birth weight—were prespecified based on their established clinical relevance to perineal trauma in the literature, rather than solely on baseline imbalance.

First- and second-degree perineal tears were analyzed as separate binary outcomes (presence vs. absence). Effect estimates were reported as odds ratios (OR) or mean differences with 95% confidence intervals (CI). Regarding the analysis of secondary outcomes, no formal adjustment for multiple comparisons (e.g., Bonferroni) was applied, as the study was primarily powered for the primary outcome; therefore, secondary results are presented as exploratory. A two-sided *p* < 0.05 was considered statistically significant.

## Results

### Participant flow

A total of 358 nulliparous pregnant women were screened for eligibility. Forty women were excluded (22 did not meet inclusion criteria, 14 declined participation, and 4 for other reasons).Thus, 318 women were enrolled (PFMT group: 161, control group: 157).

**Fig. 1:**
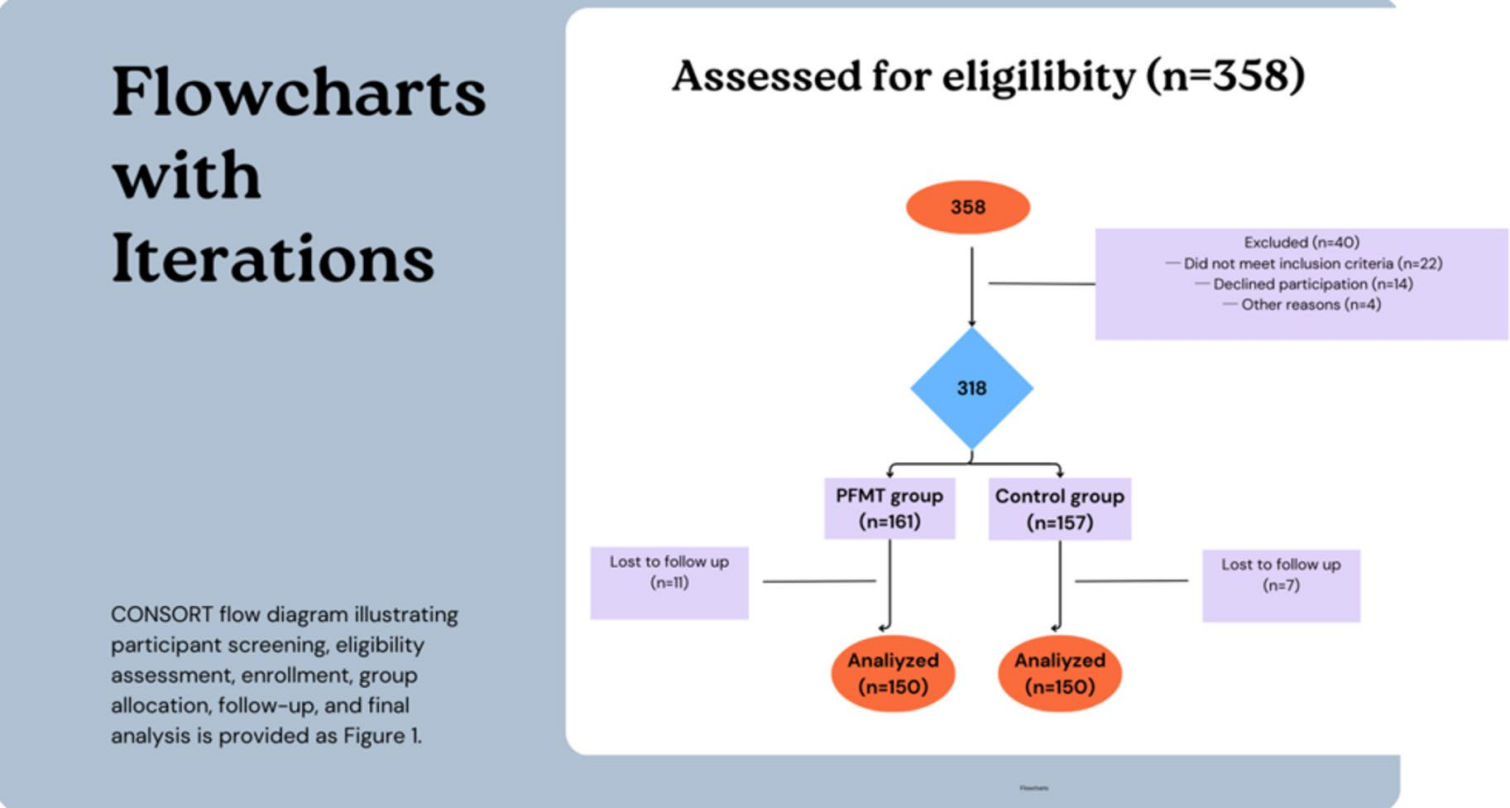
Study flow diagram.

During follow-up, 18 participants were lost (PFMT: 11, Control: 7), leaving 300 women for final analysis (PFMT: 150, Control: 150).The participant flow through each stage of the study is illustrated in Fig. 1 (Study Flow Diagram).

### Baseline characteristics

The baseline demographic and clinical characteristics of the participants are summarized in Table 1. While no statistically significant differences were observed between the PFMT and control groups regarding maternal age (*p* = 0.780), maternal weight (*p* = 0.760), or BMI (*p* = 0.712), these findings should be interpreted with caution given the non-randomized, patient-preference design. Importantly, the mean gestational age at the time of formal enrollment was comparable between groups (28.42 ± 1.15 vs. 28.51 ± 1.08 weeks; *p* = 0.485).

Regarding delivery characteristics, the majority of participants in both groups had a spontaneous labor onset (78.7% vs. 81.3%, respectively; *p* = 0.565). Rates of labor induction (21.3% vs. 18.7%, *p* = 0.565) and oxytocin augmentation (37.3% vs. 40.0%, *p* = 0.635) did not show marked imbalances between the groups. Furthermore, all participants in both groups had cephalic fetal presentation at the time of delivery.

Regarding socioeconomic status, the majority of participants in both groups had a primary school education and were housewives, reflecting a homogeneous study population. No significant differences were found in educational level (*p* = 0.814) or employment status (*p* = 0.655). Furthermore, all participants in both groups were married. The apparent similarity in neonatal birth weights (*p* = 0.420) suggests a degree of baseline comparability, although the potential for residual confounding inherent to the self-selected group allocation remains.

**Table 1:** Baseline maternal characteristics and obstetric variables.

Variable	PFMT Group (*n* = 150)	Control Group (*n* = 150)	*p*-value
Age (years), Mean ± SD	25.57 ± 5.06	25.39 ± 4.81	0.780
Weight (kg), Mean ± SD	67.00 ± 6.36	67.17 ± 6.69	0.760
BMI (kg/m²), Mean ± SD	24.82 ± 3.12	24.95 ± 3.05	0.712
Gestational Age at Enrollment (weeks)	28.42 ± 1.15	28.51 ± 1.08	0.485
Labor Onset (Spontaneous), n (%)	118 (78.7%)	122 (81.3%)	0.565
Induction of Labor, n (%)	32 (21.3%)	28 (18.7%)	0.565
Oxytocin Augmentation, n (%)	56 (37.3%)	60 (40.0%)	0.635
Fetal Presentation (Cephalic), n (%)	150 (100%)	150 (100%)	1.000
Educational Level, n (%)			0.814
• Primary School	95 (63.3%)	92 (61.3%)	
• High School	40 (26.7%)	43 (28.7%)	
• University	15 (10.0%)	15 (10.0%)	
Employment Status, n (%)			0.655
• Employed	26 (17.3%)	29 (19.3%)	
• Housewife	124 (82.7%)	121 (80.7%)	
Birth Weight (g), Mean ± SD	3269.2 ± 301.7	3226.4 ± 303.59	0.420

Note: Continuous variables were compared using independent-samples t-test (or Mann–Whitney U test if non-normal); categorical variables using chi-square or Fisher’s exact test. All participants were married (100%); therefore, no between-group comparison was performed for marital status.

### Perineal trauma and episiotomy rates

**Fig. 2:**
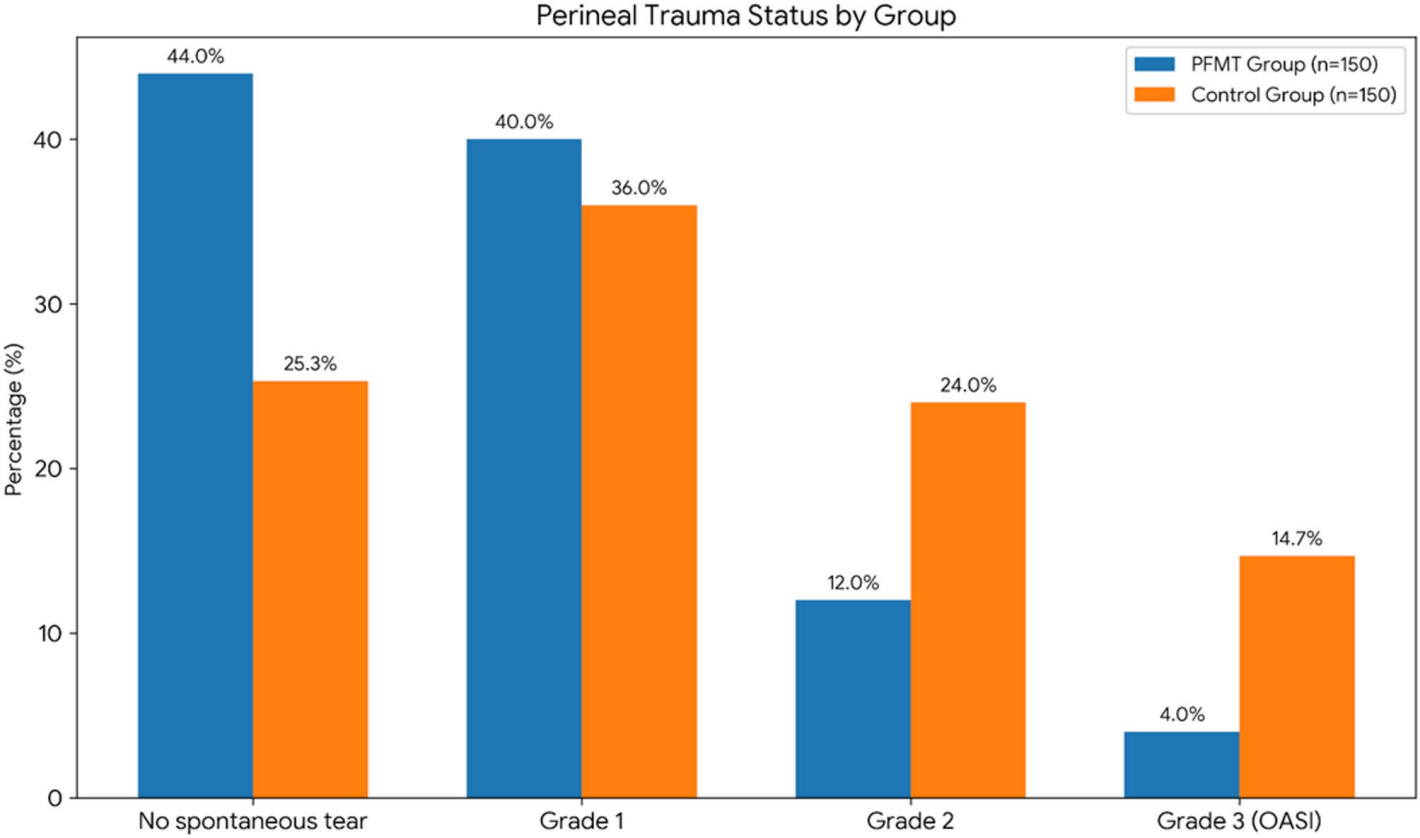
Distribution of perineal trauma grades in the PFMT and control groups.

The distribution of perineal trauma showed key differences between the groups. The incidence of severe perineal trauma (specifically third-degree tears) was significantly lower in the PFMT group compared with the control group (4.0% vs. 14.7%; unadjusted OR 0.24, 95% CI 0.09–0.64, *p* = 0.009). After adjusting for potential confounders including maternal age, BMI, and neonatal birth weight in a multivariable logistic regression model, the protective effect of PFMT remained significant (adjusted OR [aOR] 0.26, 95% CI 0.10–0.68, *p* = 0.011). This finding is illustrated in Fig. 2 and presented with adjusted estimates in Table 2.

Regarding other outcomes, the episiotomy rate was 64.0% in the PFMT group and 74.7% in the control group (unadjusted OR 0.60, 95% CI 0.36–1.01, *p* = 0.157). Multivariable analysis for episiotomy also showed no significant difference after adjustment (aOR 0.64, 95% CI 0.38–1.08, *p* = 0.192). Similarly, the prevalence of postpartum urinary incontinence at 6 weeks was not statistically different between groups, even after adjustment for baseline maternal characteristics.

### Urinary incontinence

At the 6-week postpartum follow-up, urinary incontinence of any severity was reported by 14/150 (9.3%) women in the PFMT group and 22/150 (14.7%) women in the control group (Table 2).

Most cases were classified as mild (PFMT: 8, Control: 12), while two cases of severe incontinence were reported, both in the control group. Although numerically lower in the PFMT group, this difference did not reach statistical significance (*p* = 0.645). These findings suggest a trend toward benefit that warrants confirmation in larger trials with extended follow-up. No operative vaginal deliveries (vacuum/forceps) occurred in either group.

**Table 2:** Perineal Trauma, Episiotomy, and Urinary Incontinence Outcomes.

Variable	PFMT Group (*n* = 150)	Control Group (*n* = 150)	Total (*n* = 300)	*p*-value	Adjusted OR (95% CI) †
Perineal trauma status (spontaneous tears), n (%)				0.009*	
No spontaneous tear‡	66 (44.0%)	38 (25.3%)	104 (34.7%)		
Grade 1	60 (40.0%)	54 (36.0%)	114 (38.0%)		
Grade 2	18 (12.0%)	36 (24.0%)	54 (18.0%)		
Grade 3 (OASI)	6 (4.0%)	22 (14.7%)	28 (9.3%)		0.26 (0.10–0.68)
Grade 4	0 (0.0%)	0 (0.0%)	0 (0.0%)		-
Episiotomy, n (%)				0.157	
Present	96 (64.0%)	112 (74.7%)	208 (69.3%)		0.64 (0.38–1.08)
Absent	54 (36.0%)	38 (25.3%)	92 (30.7%)		
Postpartum urinary incontinence (6 weeks), n (%)				0.645	-
None	136 (90.7%)	128 (85.3%)	264 (88.0%)		
Mild	8 (5.3%)	12 (8.0%)	20 (6.7%)		
Moderate	6 (4.0%)	8 (5.3%)	14 (4.7%)		
Severe	0 (0.0%)	2 (1.3%)	2 (0.7%)		
Duration of Second Stage of Labor (min)	49.72 ± 6.42	58.84 ± 20.42	54.28 ± 16.14	**< 0.001**	**-**
Neonatal Outcomes (Mean ± SD)					-
Apgar Score (1 min)	8.31 ± 0.47	8.23 ± 0.53	8.27 ± 0.50	0.472	
Apgar Score (5 min)	8.77 ± 0.54	8.67 ± 0.54	8.72 ± 0.54	0.538	

Note: Continuous variables are presented as mean ± SD; categorical variables as n (%). * Statistically significant at *p* < 0.05. † Adjusted Odds Ratios (aOR) were calculated using multivariable logistic regression, adjusting for maternal age, BMI, and neonatal birth weight. ‡ “No spontaneous tear” indicates absence of spontaneous tearing; this category may include women who underwent episiotomy without an additional spontaneous tear.

### Second stage of labor

The mean duration of the second stage of labor was significantly shorter in the PFMT group (49.7 ± 6.4 min) compared with the control group (58.8 ± 20.4 min).

**Fig. 3:**
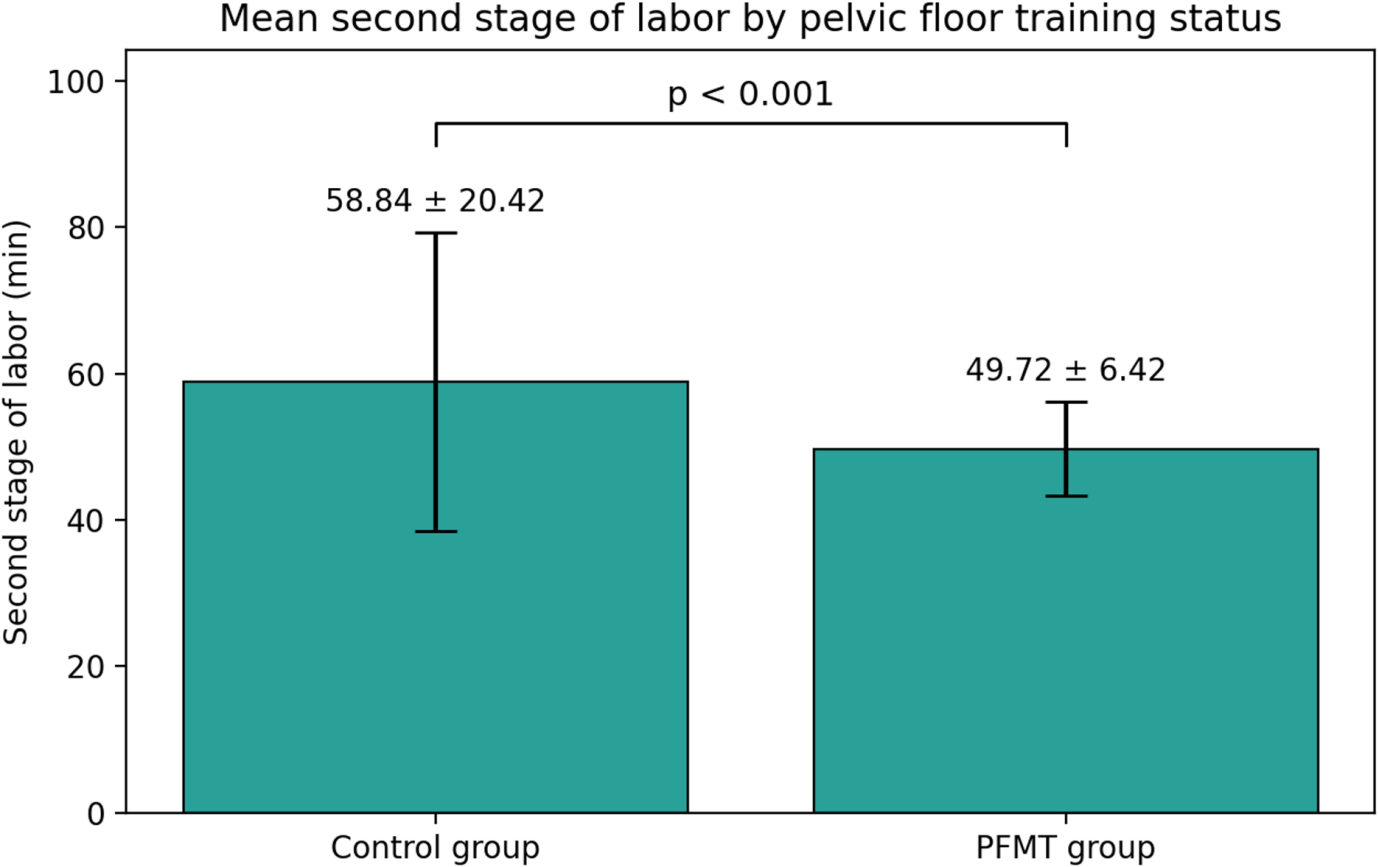
Comparison of the duration of the second stage of labor between the PFMT and control groups.

The mean difference was − 9.1 min (95% CI − 12.4 to − 5.8, *p* < 0.001), which is both statistically and clinically significant. The PFMT group also showed a narrower distribution of second-stage duration, suggesting more consistent and efficient labor progress. These findings are visually depicted in Fig. 3, which demonstrates a left-shifted distribution curve for the PFMT group.

### Neonatal outcomes

Neonatal outcomes were similar between groups (Table 2). Mean 1- and 5-minute Apgar scores and mean birth weights were comparable (*p* > 0.05).When analyzed categorically, the number of neonates with a 5-minute Apgar < 7 was very low and not significantly different between groups (PFMT: 1 [0.7%] vs. Control: 2 [1.3%], *p* = 0.621), indicating no adverse effect of PFMT on neonatal well-being.

## Discussion

### Principal findings

In this patient-preference controlled clinical trial, we found that a structured antenatal pelvic floor muscle training (PFMT) program was associated with a lower incidence of severe perineal trauma and a shorter duration of the second stage of labor, without evidence of adverse neonatal outcomes. In contrast, we observed no statistically significant between-group differences in episiotomy rates or postpartum urinary incontinence at 6 weeks. Taken together, these findings suggest a potential role for PFMT in improving selected obstetric outcomes in nulliparous women; however, causal inference is limited by the non-randomized, self-selected group allocation.

### Comparison with previous literature

Our results align with and extend previous evidence. The systematic review by Du et al. reported that antenatal PFMT was associated with a reduction in severe perineal trauma and a tendency toward shorter labor duration, although heterogeneity was noted across trials^8^. Similarly, Sobhgol et al. reported that PFMT may positively influence maternal birth outcomes but emphasized the need for further well-designed studies^9^. The Cochrane review concluded that antenatal PFMT is effective for preventing urinary incontinence in late pregnancy and early postpartum, whereas its effect on perineal trauma remains less certain due to limitations in the available high-quality data^10^. These findings are consistent with the updated Cochrane review by Boyle et al., which reinforced the role of PFMT in improving continence outcomes in antenatal and postnatal women^17^.

This study adds region-specific evidence from a Middle Eastern population. Given the patient-preference design, our findings may be most applicable to women who are willing to participate in structured PFMT; confirmation in randomized trials and in settings with different intrapartum practices is warranted before recommending broad implementation as routine care.

### Interpretation of key findings

In this patient-preference controlled clinical trial, we found that a structured antenatal PFMT program was associated with a lower incidence of severe perineal trauma and a shorter duration of the second stage of labor. Crucially, the observed associations for severe perineal trauma remained consistent after adjusting for potential confounders, including maternal age, BMI, and neonatal birth weight. The robustness of these findings after multivariable adjustment supports the hypothesis that PFMT independently contributes to improved perineal outcomes, rather than the results being driven primarily by baseline maternal characteristics.

The observed reduction in obstetric anal sphincter injuries (OASI) is important, as severe perineal trauma is associated with long-term anal incontinence, dyspareunia, and pelvic organ prolapse^6,18^. PFMT may improve pelvic floor muscle awareness and coordination, which could contribute to more controlled expulsive efforts during birth. Nevertheless, because participants self-selected into groups, residual confounding and differences in health behaviors may partly explain the observed association. In exploratory analyses, severe perineal trauma events within the PFMT group occurred in the presence of established obstetric risk factors and intrapartum determinants of anal sphincter injury. Given the low number of severe trauma events in the PFMT arm, these observations should be interpreted descriptively and considered hypothesis-generating.

The baseline OASI rate of 14.7% observed in our control group is higher than those typically reported in Western populations. This can be attributed to several factors, including our setting as high-volume tertiary referral centers. Furthermore, the high prevalence of routine mediolateral episiotomy in our setting (74.7% in the control group) may paradoxically contribute to the extension of perineal trauma into the anal sphincter^19^.

We found no statistically significant difference in episiotomy rates between groups. In settings with high baseline episiotomy use, episiotomy practice may be a dominant determinant of perineal outcomes, potentially attenuating the observable effect of PFMT on perineal injury patterns. Importantly, episiotomy should not be interpreted as protective against severe perineal trauma, and international guidance supports restrictive rather than routine use, as emphasized by the World Health Organization^20^. Because episiotomy is an iatrogenic incision and does not represent a spontaneous tear, we recorded it separately and interpret perineal trauma patterns in light of this practice context.

We also observed a shorter second stage of labor in the PFMT group, with a mean difference of approximately 9 min. While this reduction is statistically significant, its clinical meaningfulness warrants careful consideration. In routine obstetric practice, a 9-minute shortening may appear modest; however, it can be clinically relevant by reducing maternal exhaustion and potentially minimizing the period of maximal fetal head compression. This finding is consistent with prior trials and systematic reviews reporting reductions in second-stage duration^8,9,11^, and suggests more efficient labor progress contributing to a more positive birth experience.

Postpartum urinary incontinence at 6 weeks did not differ significantly between groups^10,21^. Longer-term follow-up is needed to determine whether antenatal PFMT influences urinary symptoms beyond the early postpartum period. Finally, no fourth-degree lacerations occurred in either group. Future studies in settings with more restrictive episiotomy practice may help clarify the relationship between PFMT and the full spectrum of perineal outcomes^22^.

## Strengths and limitations

### Strengths

This study is among the largest controlled clinical evaluations of antenatal pelvic floor muscle training (PFMT) conducted in a Middle Eastern population, providing region-specific evidence in an area where data remain limited^23^. The intervention was delivered using a structured and supervised protocol informed by the CERT-PFMT and TIDieR reporting frameworks, which supports transparency and reproducibility. Supervision is a major strength, as supervised PFMT programs have consistently shown superior effectiveness compared with unsupervised regimens. In addition, outcome assessors were blinded to group allocation, which reduces the risk of detection bias^24^. Finally, the direction of our findings is consistent with recent high-quality trials suggesting that PFMT-based programs particularly when combined with adjunctive approaches may yield incremental benefits^25^.

### Limitations

This study has several limitations. First, the patient-preference (non-randomized) design, while pragmatic and ethically acceptable, may introduce selection bias and residual confounding; therefore, causal inference is limited and the findings should be interpreted as associations^26^. Nevertheless, the patient-preference design is also a strength of this study in terms of ecological validity, as it aligns the intervention with maternal motivation, thereby ensuring high compliance and reflecting practical clinical outcomes. Second, the trial was registered retrospectively on ClinicalTrials.gov (NCT07292948); however, the protocol and outcomes were specified prior to recruitment and the analysis plan was finalized before database lock^27^. Third, the high baseline rate of routine mediolateral episiotomy in our setting may have influenced the distribution of spontaneous perineal tears and may have attenuated observable differences in perineal outcomes attributable to PFMT^28^. Fourth, the study was primarily powered for severe perineal trauma (OASI); analyses of secondary outcomes, including postpartum urinary incontinence and episiotomy, may have been underpowered to detect smaller yet clinically relevant effects. Fifth, adherence to the home-based component was monitored using weekly telephone calls and exercise diaries, which are subject to self-report and social desirability bias, and objective verification of home exercise performance was not available^29^. Finally, follow-up was limited to 6 weeks postpartum and longer-term pelvic floor outcomes (for example, pelvic organ prolapse, chronic pelvic floor dysfunction, and sexual function) were not assessed. Future randomized studies with longer follow-up and objective adherence measures are warranted^30^.

### Clinical implications and future directions

Our findings suggest that structured antenatal PFMT is a safe and non-invasive intervention that may reduce severe perineal trauma and shorten the second stage of labor without compromising neonatal outcomes. While these data are promising, the potential integration of such programs into routine practice could be explored, as it may be effectively achieved by utilizing existing hospital-based staff within the public healthcare infrastructure, ensuring the sustainability of the intervention without requiring additional financial resources from the patients. Integrating PFMT into standard antenatal education could be considered as a supportive adjunct strategy to improve maternal outcomes, particularly in settings with a high burden of perineal morbidity; however, the non-randomized patient-preference design warrants cautious interpretation regarding causality^31^.

Future research should focus on adequately powered randomized controlled trials with longer follow-up periods, objective adherence monitoring, and evaluation of broader outcomes such as maternal quality of life, sexual function, and cost-effectiveness. In line with recent evidence that PFMT can improve quality of life among women with pelvic floor dysfunction, future studies should include patient-reported outcomes as key endpoints and assess whether benefits persist beyond the early postpartum period^32^.

## Conclusion

Structured antenatal pelvic floor muscle training **may reduce** severe perineal trauma and **shorten** the second stage of labor in nulliparous women without adversely affecting neonatal outcomes. Although reductions in episiotomy rates and postpartum urinary incontinence did not reach statistical significance, the observed trends favor PFMT.

These findings **may support** the integration of PFMT into routine antenatal care programs and highlight the need for larger randomized trials with longer follow-up to confirm these benefits and evaluate long-term pelvic floor function.

## Data Availability

The datasets generated and analysed during the current study are available from the corresponding author on reasonable request.

## Declarations

### Previous Presentation

Preliminary results of this study were presented as an oral presentation at the KSDF 2024 Federation Congress, held on April 17–21, 2024, in Antalya, Turkey.
